# Feasibility of a Web-Based Implementation Intervention to Improve Child Dietary Intake in Early Childhood Education and Care: Pilot Randomized Controlled Trial

**DOI:** 10.2196/25902

**Published:** 2021-12-15

**Authors:** Courtney Barnes, Sze Lin Yoong, Nicole Nathan, Luke Wolfenden, Taya Wedesweiler, Jayde Kerr, Dianne S Ward, Alice Grady

**Affiliations:** 1 Hunter New England Population Health Newcastle Australia; 2 School of Medicine and Public Health University of Newcastle Callaghan Australia; 3 Hunter Medical Research Institute New Lambton Australia; 4 Priority Research Centre for Health Behaviour University of Newcastle Callaghan Australia; 5 School of Health Sciences Swinburne University of Technology Melbourne Australia; 6 Department of Nutrition, Gillings School of Global Public Health, University of North Carolina Chapel Hill, NC United States; 7 Center for Health Promotion and Disease Prevention, University of North Carolina Chapel Hill, NC United States

**Keywords:** childcare center, web-based, nutrition, healthy eating, randomized controlled trial, intervention, implementation

## Abstract

**Background:**

Internationally, the implementation of evidence-based healthy eating policies and practices within early childhood education and care (ECEC) settings that encourage children’s healthy diet is recommended. Despite the existence of evidence-based healthy eating practices, research indicates that current implementation rates are inadequate. Web-based approaches provide a potentially effective and less costly approach to support ECEC staff with implementing nutrition policies and practices.

**Objective:**

The broad aim of this pilot randomized controlled trial is to assess the feasibility of assessing the impact of a web-based program together with health promotion officer (HPO) support on ECEC center implementation of healthy eating policies and practices. Specifically, we seek to describe the completion rate of study evaluation processes (participant consent and data collection rates); examine ECEC center uptake, acceptability, and appropriateness of the intervention and implementation strategies; understand the potential cost of delivering and receiving implementation support strategies; and describe the potential impact of the web-based intervention on the implementation of targeted healthy eating practices among centers in the intervention group.

**Methods:**

A 6-month pilot implementation trial using a cluster-randomized controlled trial design was conducted in 22 ECEC centers within the Hunter New England region of New South Wales, Australia. Potentially eligible centers were distributed a recruitment package and telephoned by the research team to assess eligibility and obtain consent. Centers randomly allocated to the intervention group received access to a web-based program, together with HPO support (eg, educational outreach visit and local technical assistance) to implement 5 healthy eating practices. The web-based program incorporated audit with feedback, development of formal implementation blueprints, and educational materials to facilitate improvement in implementation. The centers allocated to the control group received the usual care.

**Results:**

Of the 57 centers approached for the study, 22 (47%) provided consent to participate. Data collection components were completed by 100% (22/22) of the centers. High uptake for implementation strategies provided by HPOs (10/11, 91% to 11/11, 100%) and the web-based program (11/11, 100%) was observed. At follow-up, intervention centers had logged on to the program at an average of 5.18 (SD 2.52) times. The web-based program and implementation support strategies were highly acceptable (10/11, 91% to 11/11, 100%). Implementation of 4 healthy eating practices improved in the intervention group, ranging from 19% (2/11) to 64% (7/11).

**Conclusions:**

This study provides promising pilot data to warrant the conduct of a fully powered implementation trial to assess the impact of the program on ECEC healthy eating practice implementation.

**Trial Registration:**

Australian New Zealand Clinical Trials Registry (ANZCTR) ACTRN12619001158156; https://www.anzctr.org.au/Trial/Registration/TrialReview.aspx?id=378099

**International Registered Report Identifier (IRRID):**

RR2-10.1186/s40814-020-00707-w

## Introduction

### Background

Poor dietary intake in early childhood, including inadequate intake of fruit and vegetables and excessive intake of discretionary foods (high in added sugar, sodium, and saturated fat), is a leading contributor to the development of childhood overweight, obesity, cardiovascular disease, and specific types of cancers [1,2]. Globally, preschool aged children do not meet national dietary recommendations for intake of fruit and vegetable servings, while overconsuming discretionary food items [2-5]. As dietary behaviors developed during childhood are known to track into adulthood [6], population-level interventions (ie, interventions targeting a large proportion of the population) to improve child nutrition are recommended [7,8]. Early childhood education and care (ECEC) is a promising setting for interventions aimed at improving children’s nutrition behaviors, as they provide access to a large proportion of children [3,9] for prolonged periods [10] during a crucial period of development [11].

Systematic review evidence has identified numerous ECEC-based interventions effective in improving child nutrition behaviors [12] and center nutrition environments [13], including the implementation of evidence-based ECEC practices associated with improved child dietary intake in care [13,14]. The implementation of such evidence-based practices is recommended within national and international ECEC guidelines and includes the provision of healthy foods, positive educator feeding practices (eg, role modeling healthy food choices), and developing center nutrition policies, which detail center strategies and guidelines to enforce the implementation of healthy eating practices [15-17]. However, despite the existence of such guidelines, numerous studies have indicated that the current implementation of evidence-based healthy eating practices is inadequate [18-21].

A recent Cochrane systematic review identified that multicomponent implementation strategies, including researcher delivered face-to-face nutrition education sessions and ongoing support, can produce small but significant improvements in the implementation of healthy eating practices in ECEC centers [13]. Although potentially effective, there are significant challenges in delivering such interventions at scale (ie, to a large number of ECEC centers), including financial and resource burden on centers and the lack of alignment with center capabilities and infrastructure [13]. Web-based modalities provide a potentially effective and less costly approach to implementing nutrition interventions at scale in this setting. Previous research suggests that the use of such modalities to deliver support to center staff is highly acceptable and fits within the existing center infrastructure (eg, access to computers and internet) [12,22,23]. In addition, these modalities can reach a large proportion of the population [24] and have been associated with improvement in a range of provider behaviors and implementation outcomes in previous research delivered outside the ECEC setting [25,26].

Recent trials examining the impact of web-based interventions on ECEC healthy eating practices have been conducted within menu-based centers (ie, centers that provide food to children). A randomized controlled trial (RCT) conducted in 54 Australian childcare centers evaluated the impact of a web-based menu planning program on center compliance with sector dietary guidelines [27]. Results of the RCT found statistically significant improvements in the servings of core food groups and child diet intake; however, the intervention had nonsignificant improvements in the primary outcome of menu compliance with all food groups. The study reported variable levels of engagement with the web-based program, despite the high uptake of implementation support strategies and high acceptability of the intervention and implementation support provided [27]. In addition, the web-based intervention was deemed a cost-effective alternative to traditional menu planning approaches [23]. Within the United States, a pilot RCT conducted in 31 centers evaluated the impact of the web-based Nutrition and Physical Activity Self-Assessment for Child Care (Go-Nutrition and Physical Activity Self-Assessment for Child Care [Go-NAPSACC]) program on center nutrition environments [28]. Despite improvements in food and beverages provided within intervention centers, no statistically significant differences in center nutrition environments were reported at follow-up [28]. Center engagement with the web-based program was not reported; however, the uptake of the implementation support strategies was high among intervention centers. Findings from the process evaluation indicated that a lack of computer literacy among center staff and the need for additional technical support were barriers to program use [28]. Despite these studies showing promise, no RCTs examining the impact of web-based interventions on ECEC healthy eating practices within lunchbox centers (ie, where parents pack foods for children to consume in care) have been conducted.

### Objectives

Given the differences between menu-based and lunchbox centers, there is a need to understand whether such interventions are feasible in the ECEC setting. Feasibility studies are recommended as they allow researchers to collect data to determine whether an intervention is appropriate for more robust testing and to pilot-test recruitment and data collection methods and tools to inform a larger trial [29]. Thus, the aim of this pilot RCT is to determine the feasibility of conducting a fully powered implementation trial assessing the impact of a web-based program together with health promotion officer (HPO) support, on childcare center implementation of healthy eating policies and practices. Specifically, we seek to (1) describe the completion of study evaluation processes (participant consent and data collection rates); (2) examine ECEC center uptake, acceptability, and appropriateness of the intervention and implementation strategies; (3) understand the potential cost of delivering and receiving the implementation strategies; and (4) describe the potential impact of the web-based intervention on the implementation of healthy eating practices among centers in the intervention group.

## Methods

### Registration and Ethics Approval

This trial was prospectively registered with the Australian New Zealand Clinical Trials Registry (ACTRN12619001158156) and followed the CONSORT reporting guidelines for pilot and feasibility studies [30]. Ethical approval for the trial was obtained from Hunter New England (HNE; HNE approval 06/07/26/4.04) and the University of Newcastle (approval H-2008-0343) Human Research Ethics Committees.

This trial was originally designed as a cluster RCT using an effectiveness-implementation hybrid type-II design. A hybrid effectiveness-implementation design was used to pilot the potential impact and assess the feasibility of an implementation intervention, while assessing the effectiveness of the intervention in improving child dietary intake in care as described by Curran et al [31]. Owing to COVID-19 precluding center site visits to conduct follow-up data collection, we were unable to undertake child lunchbox and dietary assessments and, as such, have not been reported. Therefore, this paper reports on the pilot implementation outcomes that could still be evaluated at follow-up and were specified in the trial registration and protocol.

### Study Design and Setting

A protocol detailing the study design and methodology has been published elsewhere [32]. In brief, a pilot implementation trial using a cluster RCT design was conducted in center-based childcare centers within the HNE region of New South Wales, Australia. The HNE region is socioeconomically and geographically diverse, encompassing metropolitan, regional, and remote communities, with a population of over 920,000 residents [33]. Approximately 422 center-based childcare centers, including preschools and long day care, are located within the HNE region, which typically enroll children aged 0-6 years for an average of 21 hours per week [10,34].

### Participant Eligibility and Recruitment

#### Centers

Centers were eligible to participate in the trial if (1) they enrolled >20 children per day, (2) had internet access, (3) parents provided food for children to consume while attending care (ie, centers did not provide food), (4) they did not participate in any other healthy eating or physical activity intervention, and (5) they were not fully compliant with healthy eating practices (ie, not implementing all 5 practices) specified in the NSW state obesity-prevention program (ie, *Munch & Move*) targeted by the intervention, according to the NSW Ministry of Health data monitoring [35]. Centers were ineligible if they were a mobile preschool or family day care center, did not cater to children aged 2-5 years, catered exclusively for children requiring specialist care, or were classified as an NSW Department of Education center owing to differing operational characteristics.

A list of potentially eligible centers located within the HNE region was obtained from the NSW Ministry of Health [35]. One member of the research team with experience recruiting centers to health promotion trials led the recruitment process and monitored consent rates. First, centers were progressively distributed a recruitment package consisting of a study information statement and consent form in random order. Second, the research team member leading recruitment telephoned centers to discuss study details, assess eligibility, and request consent for study participation [19,36]. The centers continued to be contacted until the required number (n=22) consented. During the telephone call, the research team member also scheduled a 2-day baseline data collection site visit for consenting centers. Recruitment for the study was conducted between August 2019 and October 2019.

#### Children

For children to be eligible to participate, they were required to (1) have written consent from a parent or guardian, (2) be between the ages of 2 and 5 years, (3) be enrolled to attend the center on at least one of the scheduled days of data collection, and (4) not have a dietary restriction requiring specialized tailoring of their diet (eg, allergies or intellectual or physical disability).

Approximately 2 weeks before the baseline data collection site visit, centers were asked to distribute consent forms and information statements to parents via usual communication methods, including email, communication apps, and child pigeonholes. Trained research assistants with experience in recruitment and data collection attended the childcare centers approximately one week before the site visit and on the days of the site visits to request written consent from parents for their children to participate in the study.

### Randomization and Blinding

Following baseline data collection, centers were randomly allocated to the intervention or control group, stratified by center socioeconomic status (SES). On the basis of center postcodes, the 2016 Socio-Economic Indexes for Areas was used to classify centers as being located in the least disadvantaged (high SES) or most disadvantaged (low SES) areas [37]. Center postcodes ranked in the top 50% of NSW were classified as least disadvantaged and the lower 50% of postcodes as the most disadvantaged. The centers were also stratified by those with a high number of Aboriginal child enrollments (defined as those with >10% Aboriginal child enrollments), in a 1:1 ratio through a block randomization procedure (block sizes 2 or 4) conducted by an independent blinded statistician. Given the nature of the intervention (ie, intervention centers were provided access to a web-based program), the centers were not blinded to group allocation. Data collectors were not blinded to group allocation at follow-up.

### Intervention

The intervention aimed to improve the implementation of childcare center–level healthy eating practices. The practices targeted within the intervention are recommended by the NSW state obesity-prevention program (ie, *Munch & Move*) [17] as well as national and international guidelines, acknowledging the association between such practices and improved child dietary intake in care [15,16]. Specifically, the practices included the following:

1. Supporting families to provide healthier foods consistent with dietary guidelines: center staff within the intervention group were provided with healthy eating information and resources via the web-based program and were asked to disseminate these to families via usual center communication methods, such as mobile apps, email, and written information, twice during the intervention period. Center staff were also asked to monitor children’s lunchboxes daily for consistency with sector-specific dietary guidelines and provide feedback to parents.
2. Provision of intentional healthy eating learning experiences (eg, gardening and cooking lessons): center staff were asked to provide children with intentional healthy eating learning experiences at least twice per week.
3. Using feeding practices that support children’s healthy eating (eg, educator role modeling healthy food choices): center staff were asked to provide encouragement to children to promote healthy eating and trying new foods at every meal and snack occasion. Center staff were also asked to role model consuming healthy food choices and avoid the use of foods to encourage desired behavior.
4. Staff participating in professional development targeting healthy eating: center staff were asked to have at least 50% of the staff to participate in web-based training opportunities specific to staff healthy eating behaviors and center practices.
5. Having a comprehensive written nutrition policy that outlines key healthy eating practices: centers were asked to develop or modify existing nutrition policies to document procedures and strategies to facilitate the implementation of healthy eating practices to improve child diet.

A detailed description of these practices is provided in the study protocol [32].

A web-based program, known as Childcare Electronic Assessment Tool and Support (EATS), was developed by the research team to support center implementation of the 5 targeted healthy eating practices. The centers allocated to the intervention group were provided with free access to the web-based program. The intervention was developed by behavioral science researchers, HPOs, state government representatives, and end users from the ECEC setting, including nominated supervisors and educators.

The Behavior Change Wheel (BCW) [38] was used to guide the development and selection of implementation strategies to support center staff in achieving behavior change. During this process, barriers and enablers to center behavior change identified through a literature review and engagement with ECEC staff and stakeholders were mapped to specific behavior change techniques (BCTs) within the BCW [38]. A suite of implementation strategies, defined according to the expert recommendations for implementing change taxonomy, were then selected to action the BCTs within the intervention [39]. The content and implementation strategies within Childcare EATS were selected to ensure user (ie, center staff) engagement, including self-assessment and action planning components to allow center nominated supervisors to reflect on current practice and housed educational resources to facilitate improvements in staff behavior and center processes. The features of the program were developed to integrate within existing center procedures, (eg, the ability to download feedback from the self-assessment quiz) and national assessment and rating standards (eg, the development of action plans as evidence within quality improvement plans). Extensive pilot testing was undertaken with ECEC staff through face-to-face meetings with HPOs to ensure that the functionality and content of Childcare EATS was appropriate and that any potential barriers to program use were addressed. Limitations from previous web-based interventions conducted within the ECEC setting, including low staff computer literacy, need for ongoing technical support, and competing priorities of ECEC staff were also considered during the development of the program [28,40].

Implementation strategies additional to those embedded within the web-based program identified via the BCW process above were used by HPOs who work within the state local health districts to deliver health promotion initiatives within community-based settings such as childcare centers. The HPOs received a training session and implementation manual before delivering the intervention. In addition, HPOs conducted 2 pilot training sessions, with both internal (health service staff with extensive experience supporting ECEC centers to implement obesity prevention initiatives) and external (ECEC center staff) stakeholders. The application of these implementation strategies within the intervention is summarized in Table 1 using the Proctor framework [41] to enable replication.

**Table 1:** Implementation strategies and behavior change techniques used within the web-based intervention.

Mode of delivery and implementation strategy according to ERIC^a^ [39]			Application of the implementation strategy according to Proctor [41]		Behavior change technique actioned via the implementation strategy
**Web-based program**					
	Audit with feedback	Actor: web-based program Action: the Childcare EATS^b^ program contained a self-assessment feature for centers to assess implementation of targeted healthy eating practices. Centers were automatically provided with tailored feedback on practice performance. Target: nominated supervisors and center champion knowledge, behavior and abilities, perceived capabilities, and confidence to implement change Temporality: commencement of the intervention. Centers were encouraged to complete the self-assessment at least twice during the intervention period to monitor change in practice, following the educational outreach visit. Dose: twice during the intervention period Implementation outcome: implementation of healthy eating practices Justification: provision of feedback on center behavior has been used within previous interventions to facilitate improvement in practice within ECEC^c^ centers [28,42]		Feedback on behavior Feedback on outcome of behavior Self-monitoring of behavior	
	Develop a formal implementation blueprint	Actor: web-based program Action: following the completion of self-assessment, centers were encouraged to select goals and develop an action plan within the Childcare EATS program. Target: nominated supervisors and center champion prioritization and investment and perceived capabilities to implement change; formalized guidance and demonstrated support to implement change Temporality: commencement of the intervention. Centers were encouraged to develop an action plan at least twice within the intervention period, immediately following the self-assessment (audit with feedback). Dose: twice during the intervention period Implementation outcome: implementation of healthy eating practices Justification: developing a formal implementation blueprint has been used within previous interventions to facilitate improvement in practice within ECEC centers [28].		Goal-setting (outcome and behavior) Action planning Problem solving Review goals (outcome and behavior)	
	Distribute educational materials	Actor: web-based program Action: the Childcare EATS program housed a suite of materials to assist center implementation of the targeted practices, including factsheets and resources to facilitate communication with parents; educational materials to improve staff knowledge; example healthy eating learning experiences; professional development and policy templates. Target: nominated supervisors and center champions to increase staff member knowledge and abilities to implement practices Temporality: commencement of the intervention. Centers were encouraged to access resources immediately following action planning (development of a formal implementation blueprint). Dose: accessed at any time during the intervention period Implementation outcome: implementation of healthy eating practices Justification: the provision of support and resources via web-based programs is highly acceptable among ECEC staff and has been used within previous interventions within the ECEC setting [22,27,28].		Demonstration of behavior Restructuring the physical environment Adding objects to the environment Prompts or cues Credible source	
**Health promotion officer**					
	Educational outreach visit	Actor: HPO^d^ Action: 1.5-2–hour practical face-to-face training session with an HPO was provided to nominated supervisors and center champions to introduce the web-based program and support implementation of the healthy eating practices. Target: nominated supervisors and center champion knowledge and ability to implement change Temporality: one-off face-to-face training session (1.5-2 hours) at the start of the intervention (2-8 weeks after baseline) Dose: one-off training session Implementation outcome: adoption of the intervention Justification: face-to-face training within previous ECEC-based interventions has been highly acceptable and used within previous interventions conducted by the research team [27,42]		Instruction on how to perform behavior Demonstration on how to perform behavior	
	Identify and prepare a center champion	Actor: center champion Action: center nominated supervisors were asked to identify and prepare a staff member who could dedicate themselves to endorsing and driving implementation of the intervention within their center and asked to attend the educational outreach visit. Target: center champions; staff investment and motivation to change, formalized guidance and demonstrated support for staff Temporality: commencement of the intervention period Dose: ongoing endorsement and support for use of the web-based program throughout the intervention period Implementation outcome: adoption of the intervention and implementation of healthy eating practices Justification: preparing a champion has been identified as an effective strategy to drive implementation and has been used in previous trials by the research team [39,43,44].		Identification of self as role model Social support (unspecified)	
	Mandate change	Actor: HPO, nominated supervisor, and center champion Action: an MoU^e^ was developed to outline the responsibilities and level of commitment expected from both the center and the HPO in working to implement the targeted healthy eating practices. Center nominated supervisors and champions discussed the MoU with the HPO and tailored the content of the MoU to suit the needs of the center. Target: nominated supervisors and center champion investment and motivation to change, formalized guidance and demonstrated support for staff Temporality: MoU drafted during the face-to-face educational outreach visit and finalized and signed by the nominated supervisor, center champion, and HPO 2 weeks following the training Dose: one-off MoU during the face-to-face educational outreach visit, followed by ongoing advocating and support for use of the web-based program by the nominated supervisor and center champion to center staff during the intervention period Implementation outcome: adoption of the intervention Justification: securing executive support from nominated supervisors has been effective in improving implementation of healthy eating practices in previous ECEC-based interventions [19]		Commitment Social support (unspecified)	
	Ongoing consultation and local technical assistance	Actor: HPO Action: a telephone call was provided to nominated supervisors and center champions to discuss barriers to center implementation of healthy eating practices and the use of the Childcare EATS program, and to develop strategies to address such barriers. Email and telephone support was provided by HPOs upon center request. Target: nominated supervisors and center champion prioritization and confidence to implement change, formalized guidance, and support Temporality: 1 telephone call made to centers approximately 2 months following the face-to-face training session Dose: once during the intervention period Implementation outcome: adoption of the intervention and implementation of healthy eating practices Justification: ongoing consultation has been shown to be effective in improving implementation, staff motivation and problem solving within ECEC-based interventions [45,46].		Social support (unspecified) Verbal persuasion about capability	

^a^ERIC: expert recommendations for implementing change.

^b^EATS: Electronic Assessment Tool and Support.

^c^ECEC: early childhood education and care.

^d^HPO: health promotional officers.

^e^MoU: memorandum of understanding.

### Control

Centers allocated to the control group received usual care during the intervention period, including general support from HPOs external to the research team upon request to implement the NSW state obesity-prevention program (ie, *Munch & Move*). The provision of such support was centrally monitored by the research team, with 1 center receiving educational materials to support the implementation of healthy eating and physical activity practices before baseline data collection.

### Data Collection and Measures

Baseline data were collected between September 2019 and December 2019, and follow-up data were collected between September 2020 and October 2020. A summary of the study outcomes and time points of measurement is provided in Table 2.

**Table 2:** Study outcomes and time points of measurement.

Study outcome		Time points of measurement
Center and child demographics		Baseline
**Feasibility of the evaluation procedures**		
	Childcare center and child consent rates	Baseline
	Completion of data collection components	Baseline
**Uptake, acceptability, and appropriateness of the intervention and implementation strategies**		
	Delivery of the implementation strategies	6 months
	Engagement with the Childcare EATS^a^ web-based program	6 months
	Acceptability of the implementation strategies	12-month follow-up
	Appropriateness of the intervention	12-month follow-up
Cost of implementation strategy delivery		Continuously across study period
Implementation of targeted healthy eating practices within the intervention group		Baseline and 6 months

^a^EATS: Electronic Assessment Tool and Support.

### Outcomes: Center and Child Demographics

At baseline, a web-based or telephone interview (depending on center preference) with center nominated supervisors was conducted to collect center demographic information, including the type of center (ie, preschool or long day care), center operating hours, number of Aboriginal or Torres Strait Islander enrollments, and number of children enrolled aged between 2 and 5 years. Center area SES and geographic location were determined using the center postcodes. Nominated supervisor demographic information, including age, was also collected during the baseline interviews. A web-based or telephone interview (depending on center preference) was conducted with center champions at follow-up to collect demographic information, including age.

Information recorded on parent consent forms was used to examine the child demographics. Parents reported the child’s age, sex (as recorded on the child’s birth certificate), Aboriginal or Torres Strait Islander background, and usual number of days attending care.

### Feasibility of the Evaluation Procedures

The feasibility of the evaluation procedures, defined as the extent to which the research can be effectively carried out within the ECEC setting [47], was assessed via parent and center consent rates and completion of data collection components.

Childcare center and child consent rates were assessed using internal records kept by the research team, center, and child consent forms. Center consent rates were calculated as the number of consenting centers divided by the number of eligible centers that were approached to participate in the study. Reasons for centers declining to participate and ineligibility were recorded by the staff member conducting the recruitment telephone calls. Research assistants present on the days of data collection collated all returned child consent forms, including those from parents who did not provide consent for their child to participate in the study. Class lists specific to the days of data collection were obtained from each participating center to determine the total number of eligible children, with consent rates calculated as the number of consenting children divided by the total number of eligible children.

Completion of data collection components including lunchbox observations and measurements, web-based or telephone interviews with nominated supervisors, and observations of the center nutrition environments, was monitored via internal records kept by the research team. These data collection components were used to evaluate the originally planned trial outcomes related to the center nutrition environment and child dietary intake. Center completion of each individual component of data collection (web-based or telephone interview and assessment of center nutrition environments) was collated and entered into a tracking spreadsheet by a member of the research team. The number of complete child dietary intake data collection forms completed during center site visits was counted and included in the tracking spreadsheet.

### Uptake, Acceptability, and Appropriateness of the Intervention and Implementation Strategies

The delivery of the implementation strategies was monitored using internal records maintained by the research team. For each center, the following information was recorded: center receipt of each implementation strategy (ie, number of centers that were offered and accepted or declined each strategy), date, duration, and type (ie, email, telephone, or face-to-face) of each implementation strategy delivered, the role of center staff receiving the implementation strategy (ie, nominated supervisor or center champion), and the delivery of BCTs within each implementation strategy (Table 1).

Engagement with the Childcare EATS web-based program was assessed via Google Analytics [48] embedded within the program. Information collected via the analytics included center completion of self-assessments (ie, audit with feedback), development of action plans (ie, developing a formal implementation blueprint), frequency of centers accessing educational materials, total log-ins to Childcare EATS, and average duration of the log-ins. Such measures have been reported in previous ECEC web-based interventions [27,49].

The acceptability of the implementation strategies, defined as the perception among center staff that the implementation strategies are satisfactory, palatable, or agreeable [47], was assessed through web-based and telephone interviews with nominated supervisors and center champions at follow-up. Interview items were modified from those developed by Weiner et al [50] and those used by the research team in previous ECEC-based studies [27,51]. In total, 10 items captured information on the perceived effectiveness (eg, ease of use and helpful in assessing and improving implementation of practices) of the Childcare EATS web-based program and usefulness of the implementation support strategies [27,47,51]. Nominated supervisors responded to each item on a 5-point Likert scale (1=strongly agree to 5=strongly disagree), with the proportion reporting 2 or lower (agree and strongly agree) for each item calculated.

The appropriateness of the intervention, defined as the perceived fit, relevance, or compatibility of the intervention and for the childcare setting [50], was assessed during the web-based or telephone interview with nominated supervisors at follow-up. In total, 4 items captured information on the perceived fit and suitability of healthy eating practices, using modified items by Weiner et al [50]. Nominated supervisors responded to each item on a 5-point Likert scale (1=strongly agree to 5=strongly disagree), with the proportion reporting 2 or lower (agree and strongly agree) for each item calculated.

### Cost to Deliver and Receive Implementation Strategies

The direct cost of each implementation strategy delivered by HPOs, including labor (ie, HPO preparation, administration, and delivery of the strategy) and travel, was calculated. Service delivery costs were recorded by the HPOs delivering the intervention. Costs (in Aus $ and US $, 2019/2020) were calculated by multiplying the time spent (in hours) on each implementation strategy by the hourly wage rate of HPOs delivering the intervention. The cost for nominated supervisors and center champions to receive the implementation strategies delivered by HPOs and embedded within the web-based program was also calculated. Data to calculate center costs were recorded by the HPOs delivering the intervention in addition to the time spent in the web-based program captured by the analytics data. Similar to previous studies examining the cost of receiving interventions within the childcare setting [23], costs were calculated by multiplying the time spent (in hours) receiving each implementation strategy by the estimated hourly wage rate of nominated supervisors and educators [52].

### Implementation of Targeted Healthy Eating Practices Within the Intervention Group

Self-reported implementation of the 5 targeted healthy eating practices within the intervention group was assessed via baseline nominated supervisor interview data and self-assessments completed by centers via the web-based program at any time point throughout the intervention. In total, 26 items were based on the validated Environment and Policy Assessment and Observation Self-Report [53] and the tool developed by Dodds et al [54] were used to measure the implementation of the 5 healthy eating practices.

In addition, we also assessed contextual factors influencing the center implementation of healthy eating practices, assessed through web-based and telephone interviews with nominated supervisors at follow-up. A total of 5 interview items were based on constructs within 3 of the 5 domains of the Consolidated Framework for Implementation Research (inner setting: compatibility with center values and direction), innovation characteristics (perceived complexity and cost), and outer setting (external influences such as policies and regulations) to identify factors associated with implementation [55]. Nominated supervisors responded to each item on a 5-point Likert scale (1=strongly agree to 5=strongly disagree), with the proportion reporting 2 or lower (agree and strongly agree) for each item calculated.

### Statistical Analysis

All statistical analyses were performed using STATA v14 (StataCorp LLC) [56]. All data were analyzed using descriptive statistics. Chi-square analyses were used to compare characteristics of consenting and nonconsenting centers as well as center and child characteristics between the intervention and control groups at baseline. Center locality was classified as either urban (ie, major cities) or rural (ie, inner regional, outer regional, and remote) according to the Australian Statistical Geography Standard [57]. The 2016 Socio-Economic Indexes for Areas was used to classify centers as being located in the least disadvantaged (high SES) or most disadvantaged (low SES) areas [37]. Center postcodes ranked in the top 50% of NSW were classified as least disadvantaged and the lower 50% of postcodes as the most disadvantaged.

## Results

### Overview

**Figure 1:**
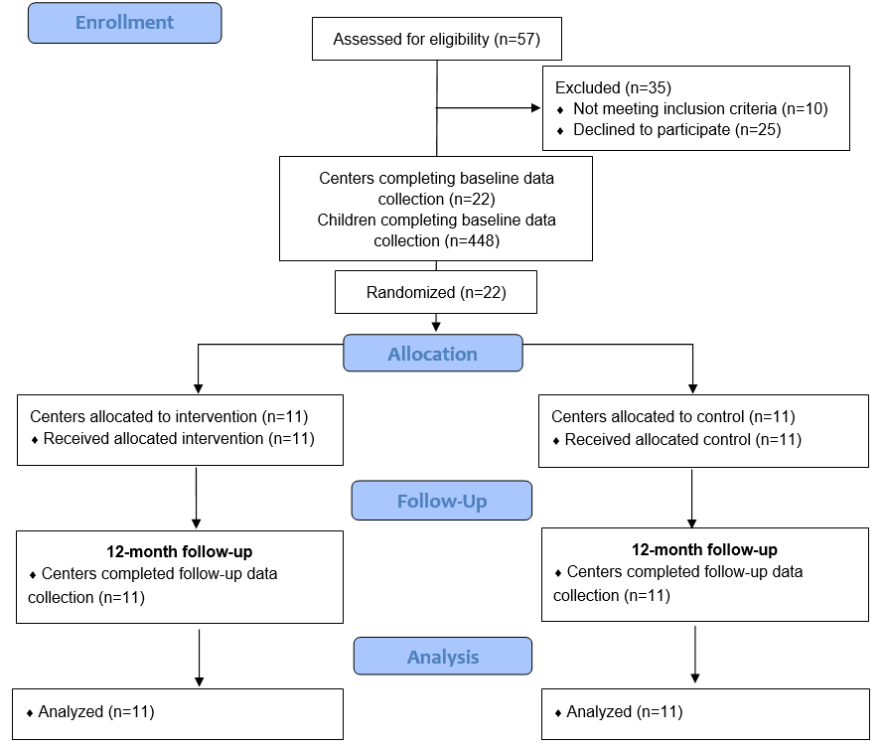
Study flow diagram.

A total of 22 centers and 448 children participated in the study, with 11 (50%) centers randomized to the intervention group and 11 (50%) to the control group (see Figure 1 for the CONSORT diagram). The demographic characteristics of consenting centers and children are summarized in Table 3. There were no significant differences in center SES or center geographic location between the consenting and nonconsenting centers. In addition, there were no significant differences in the center or child characteristics between the intervention and control groups at baseline.

**Table 3:** Demographic characteristics of participating centers and children (N=11).

Center characteristics		Intervention		Control
**Type of center**				
	Preschool, n (%)	10 (90)		10 (90)
	Long day care, n (%)	1 (10)		1 (10)
Child enrollments aged 2-5 years, mean (SD)		30.73 (11.24)		29.0 (8.63)
Aboriginal child enrollments, mean (SD)		5.0 (4.58)		4.64 (3.32)
**SEIFA^a,b^**				
	Most disadvantaged (low SES^c^), n (%)	4 (36)		4 (36)
	Least disadvantaged (high SES), n (%)	7 (64)		7 (64)
**Geographic location**				
	Urban (major cities), n (%)	8 (73)		8 (73)
	Rural (inner regional, outer regional, and remote), n (%)	3 (27)		3 (27)
**Nominated supervisor characteristics**				
	Age, mean (SD)	37.68 (5.92)		43.91 (10.57)
**Center champion characteristics, N**		6		—^d^
	Age (years), mean (SD)	44.17 (6.40)		
**Child characteristics, N**		246		202
	Age (years), mean (SD)	4.68 (0.66)		4.65 (0.68)
**Gender, N**		246	202	
	Female, n (%)	122 (49.5)		88 (43.5)
	Male, n (%)	124 (50.4)		114 (56.4)
Children of Aboriginal and Torres Strait Islander background, n (%)		24 (9.7)		20 (9.9)
Days attending care, mean (SD)		2.63 (0.88)		2.57 (0.74)

^a^SEIFA: Socio-Economic Indexes for Areas.

^b^The 2016 Socio-Economic Indexes for Areas was used to classify centers as being located in the least disadvantaged (high socioeconomic status) or most disadvantaged (low socioeconomic status) areas. Center postcodes ranked in the top 50% of New South Wales were classified as least disadvantaged and the lower 50% of postcodes as the most disadvantaged.

^c^SES: socioeconomic status.

^d^Data not available (this item was only applied to nominated supervisors).

### Feasibility of the Evaluation Procedures

#### Childcare Center and Child Consent Rates

Of the 85 potentially eligible centers within the sampling frame, 57 (67%) centers were approached in random order to participate in the study. Of the 57 centers, 10 (18%) centers were ineligible (NSW Department of Education center: 6/10, 60%; involved in another healthy eating or physical activity research trial: 1/10, 10%; and provided food to children: 3/10, 30%) and 25 (44%) centers declined to participate (lack of time: 21/25, 84%; study of lessor importance: 2/25, 8%; and lack of staff capacity: 2/25, 8%). This resulted in an overall study consent rate of 47% (22/57). No centers withdrew from the trial following randomization.

A potential 670 children were eligible to participate in the lunchbox measurements, of whom, 502 (74.9%) provided consent to participate. The consent rate ranged from 53% (16/30) to 96% (24/25) within the participating centers (285/374, 76.2% children within intervention centers and 217/296, 73.3% children within control centers).

#### Completion of Data Collection Components

Baseline lunchbox observations and measurements conducted to assess the impact of the intervention on child dietary intake were completed for 100% (448/448) of consenting children who were in attendance on data collection days at baseline. The remaining 10.8% (54/502) of the children were absent on the data collection days. Baseline observations of the nutrition environment and web-based or telephone interviews with center nominated supervisors were completed for 100% (22/22) of participating centers.

### Uptake, Acceptability, and Appropriateness of the Intervention and Implementation Strategies

#### Delivery of Implementation Strategies

For implementation strategies delivered by the HPO, 100% of center nominated supervisors or directors were offered and received the educational outreach visit (ie, face-to-face training session) with the HPO at the commencement of the intervention. The mean duration of the educational outreach visit was 92.73 (SD 21.83) minutes. All centers (n=11) were invited to nominate and prepare a staff member as center champion, with 55% (6/11) of centers nominating a staff member, and 83% (5/6) of these also attending the educational outreach visit. The memorandum of understanding (MoU; ie, mandate change) was drafted with all intervention centers (n=11), with a signed MoU returned by 55% (6/11) of the centers. Ongoing consultation and local technical assistance (ie, follow-up support call provided by the HPO) were offered to 100% (11/11) of the intervention centers, with 91% (10/11) of the centers accepting the call. The mean duration of the follow-up support call was 11.9 (SD 4.70) minutes.

For implementation strategies within the web-based program, overall, 100% (11/11) of centers were provided access to and undertook audit with feedback (ie, self-assessment), developed a formal implementation blueprint (ie, action plan), and accessed the educational materials via the Childcare EATS web-based program.

All intervention centers (n=11) received BCTs as intended in 57% (4/7) of the implementation strategies (Table 4). Additional BCTs (instruction on how to perform the behavior, problem solving, social support [practical], and action planning) were used within the ongoing consultation and local technical assistance strategy in 37% (4/11) of the centers owing to the HPO responding to the needs of the center and tailoring the advice accordingly. Low uptake of the mandate change and identification and preparation of center champion implementation strategies resulted in only 55% (6/11) of the centers receiving the BCTs within these strategies.

**Table 4:** Behavior change techniques delivered within implementation strategies (N=11).

Mode of delivery, implementation strategy, and behavior change technique			Number of centers
**Web-based program**			
	**Audit with feedback**		
		Feedback on behavior	11 (100)
		Feedback on outcome of behavior	11 (100)
		Self-monitoring of behavior	11 (100)
	**Develop a formal implementation blueprint**		
		Goal-setting (outcome and behavior)	11 (100)
		Action planning	11 (100)
		Problem solving	11 (100)
		Review goals (outcome and behavior)	11 (100)
	**Distribute educational materials**		
		Demonstration of behavior	11 (100)
		Restructuring the physical environment	11 (100)
		Adding objects to the environment	11 (100)
		Prompts or cues	11 (100)
		Credible source	11 (100)
**Health promotion officer**			
	**Educational outreach visit**		
		Instruction on how to perform behavior	11 (100)
		Demonstration on how to perform behavior	11 (100)
	**Ongoing consultation and local technical assistance**		
		Social support (unspecified)	10 (91)
		Verbal persuasion about capability	10 (91)
		Instruction on how to perform behavior^a^	3 (27)
		Problem solving^a^	1 (9)
		Social support (practical)^a^	1 (9)
		Action planning^a^	3 (27)
	**Mandate change**		
		Commitment	6 (55)
		Social support (unspecified)	6 (55)
	**Identify and prepare a center champion**		
		Identification of self as role model	6 (55)
		Social support (unspecified)	6 (55)

^a^Additional behavior change techniques used within the ongoing consultation and local technical assistance implementation strategy beyond that specified in the intervention protocol.

#### Engagement With the Web-Based Program

The intervention center’s engagement with the Childcare EATS web-based program is detailed in Table 5. At the 6-month follow-up, intervention centers had logged in to the program on an average of 5.18 (SD 2.52) times, spending an average of 19.90 (SD 11.21) minutes in the program per log-in. Centers completed an average of 2.90 (SD 2.02) self-assessments and developed an average of 2.09 (SD 1.30) action plans. A total of 6 staff members from 4 intervention centers completed web-based professional development accessible via the web-based program or the NSW state obesity-prevention program website (ie, *Munch & Move*) during the intervention period compared with no staff members from control centers.

**Table 5:** Center engagement with Childcare Electronic Assessment Tool and Support web-based program across 6 months.

Engagement	Value, mean (SD)	Value, median (IQR)
Total log-ins	5.18 (2.52)	4.00 (4.00-5.00)
Average log-in duration (minutes)	19.90 (11.21)	17.44 (10.24-30.03)
Self-assessments completed	2.90 (2.02)	2.00 (1.00-4.00)
Action plans developed	2.09 (1.30)	2.00 (1.00-3.00)
Number of times educational materials were accessed	12.36 (6.71)	10.00 (6.00-18.00)

#### Acceptability of the Intervention and Implementation Strategies

The web-based program was reported to be an acceptable method for assessing healthy eating practices by most nominated supervisors (10/11, 91%) and center champions (5/6, 83%; Table 6). The implementation strategies provided by HPOs, including the educational outreach visit (ie, face-to-face training) and ongoing support (ie, support call), were considered to be acceptable by nominated supervisors (10/11, 91% to 11/11, 100%). Acceptability of the implementation strategies was lower among center champions (2/6, 33% to 5/6, 83%).

**Table 6:** Acceptability and appropriateness of the web-based intervention and implementation strategies.

Characteristics			Nominated supervisors (n=11), n (%)		Center champions (n=6), n (%)
**Measure (agree or strongly agree)**					
	Using the web-based program is an acceptable method for assessing if our service is meeting the healthy eating policies and practices.	10 (91)		5 (83)	
	The web-based program was useful in my service to help meet the healthy eating policies and practices.	11 (100)		5 (83)	
	Using the web-based program improved my service’s performance in meeting the healthy eating policies and practices.	10 (91)		5 (83)	
	I would recommend the web-based program to other childcare services.	10 (91)		5 (83)	
	I intend to continue to use the web-based program to help our service meet the healthy eating policies and practices.	10 (91)		5 (83)	
	I thought the web-based program was easy to use.	10 (91)		—^a^	
**Measure (useful or very useful)**					
	I found the face-to-face training session (ie, educational outreach visit) useful.	10 (91)		5 (83)	
	I found the garnering of managerial support (ie, mandate change) useful.	11 (100)		2 (33)	
	I found the ongoing telephone support (ie, ongoing consultation and local technical assistance) provided by the health promotion officers useful.	10 (91)		2 (33)	
	I found nominating a center champion (ie, identify and prepare a center champion) useful.^b^	5 (83)		—	
**Appropriateness (agree or strongly agree)**			11 (100)		—
	The healthy eating policies and practices seem fitting.				
	The healthy eating policies and practices seems suitable.				
	The healthy eating policies and practices seem applicable.				
	The healthy eating policies and practices seem like a good match.				
**Contextual factors influencing implementation of healthy eating practices (agree or strongly agree)**					—
	The healthy eating policies and practices are consistent with our center philosophy.	10 (91)			
	The healthy eating policies and practices are consistent with the National Quality Framework.	10 (91)			
	The healthy eating policies and practices are costly to implement.	0 (0)			
	The healthy eating policies and practices are difficult to implement.	4 (36)			
	Centers within our region would be supportive of the healthy eating policies and practices.	10 (91)			

^a^Data not available (this item was only applied to nominated supervisors).

^b^This item was only applied to centers that nominated a center champion (n=6).

#### Appropriateness of the Intervention

In total, 100% (11/11) of nominated supervisors within the intervention group agreed or strongly agreed that healthy eating policies and practices seem fitting, suitable, applicable, and a good match (Table 6).

### Cost to Deliver and Receive Implementation Strategies

The total cost to the health service for the HPO to deliver the implementation strategies (ie, educational outreach visit, mandate change, and ongoing consultation) was Aus $ 1351.25 (US $972.64), average per center: Aus $ 122.84 (US $88.42). Overall, the educational outreach visits cost a total of Aus $ 1143.08 (US $822.79), average per center: Aus $ 103.92 (US $74.80), including travel to the center and follow-up correspondence with center staff; mandate change cost a total of Aus $ 43.44 (US $31.27), average per center: Aus $ 3.95 (US $2.84); and ongoing consultation cost a total of Aus $164.73 (US $118.57), average per center: Aus $ 14.98 (US $10.78). The total cost to centers for nominated supervisors and center champions to receive all implementation strategies (ie, those delivered by the HPO and embedded within the web-based program) was Aus $ 1516.40 (US $1091.51), average per center: Aus $ 137.85 (US $99.23). The cost to receive the implementation strategies delivered by the HPO was Aus $ 1052.29 (US $757.44), average per center: Aus $ 95.66 (US $68.86), whereas the cost to receive the implementation strategies embedded within the web-based program was Aus $ 464.11 (US $334.07), average per center: Aus $ 42.19 (US $30.37).

### Implementation of Targeted Healthy Eating Practices Within the Intervention Group

The proportion of centers implementing targeted healthy eating practices improved in 4 of the 5 practices from baseline to follow-up (Table 7). The greatest improvement was reported in center educator use of feeding practices that support children’s healthy eating, increasing from 18% (2/11) to 82% (9/11). The proportion of centers supporting families to provide healthier foods consistent with dietary guidelines decreased from 82% (9/11) to 55% (6/11). At follow-up, 18% (2/11) of centers were implementing all 5 healthy eating practices, whereas none were at baseline. The mean number of practices implemented per center increased from 3.36 (SD 1.21) at baseline to 4.36 (SD 1.21) at follow-up. When examining the change in practice implementation between the most (low SES) and least (high SES) disadvantaged centers, the number of most disadvantaged centers supporting families to provide healthier foods consistent with dietary guidelines reduced from 100% (4/4) at baseline to 25% (1/4) at follow-up compared with no change in least disadvantaged centers (Table 8).

In total, 91% (10/11) of nominated supervisors reported that healthy eating practices were consistent with the philosophy of their service and consistent with the ECEC settings regulatory standards (ie, the National Quality Framework; Table 6).

**Table 7:** Intervention group implementation of healthy eating practices (N=11).

Healthy eating practice	Centers implementing at baseline, n (%)	Centers implementing at follow-up, n (%)	Change, n (%)
Provision of intentional healthy eating learning experiences	4 (36)	6 (55)	2 (18)
Comprehensive written nutrition policy that outlines key healthy eating practices	8 (73)	10 (91)	2 (18)
Staff participating in professional development targeting healthy eating	3 (27)	6 (55)	3 (27)
Educator use of feeding practices that support children’s healthy eating	2 (18)	9 (82)	7 (64)
Supporting families to provide healthier foods consistent with dietary guidelines	9 (82)	6 (55)	−3 (27)

**Table 8:** Intervention group implementation of healthy eating practices by Socio-Economic Indexes for Areas classification (N=1)^a^.

Healthy eating practice	Low SES^b^ (n=4), n (%)				High SES (n=7), n (%)		
	Most disadvantaged centers implementing at baseline	Most disadvantaged centers implementing at follow-up	Change	Least disadvantaged centers implementing at baseline		Least disadvantaged centers implementing at follow-up	Change
Provision of intentional healthy eating learning experiences	2 (50)	2 (50)	0 (0)	2 (29)		4 (57)	2 (29)
Comprehensive written nutrition policy that outlines key healthy eating practices	3 (75)	3 (75)	0 (0)	5 (71)		7 (100)	2 (29)
Staff participating in professional development targeting healthy eating	1 (25)	1 (25)	0 (0)	1 (14)		5 (71)	4 (57)
Educator use of feeding practices that support children’s healthy eating	1 (25)	4 (100)	3 (75)	1 (14)		5 (71)	4 (57)
Supporting families to provide healthier foods consistent with dietary guidelines	4 (100)	1 (25)	−3 (75)	5 (71)		5 (71)	0 (0)

^a^The 2016 Socio-Economic Indexes for Areas was used to classify centers as being located in the least disadvantaged (high socioeconomic status) or most disadvantaged (low socioeconomic status) areas. Center postcodes ranked in the top 50% of New South Wales were classified as least disadvantaged and the lower 50% of postcodes as the most disadvantaged.

^b^SES: socioeconomic status.

## Discussion

### Principal Findings

This study aimed to assess the potential feasibility of a pilot cluster RCT of a web-based healthy eating implementation intervention in ECEC centers to undertake a fully powered implementation trial. The study also examined the uptake, acceptability, appropriateness, and actual cost of delivering the intervention and implementation strategies. Overall, the study findings indicate that the web-based intervention and most implementation strategies are highly feasible, low-cost, and acceptable to childcare center staff and can improve the implementation of healthy eating practices in ECEC centers.

The study obtained a high overall parental consent rate of 74.9% (502/670) for children to participate in lunchbox measurements. However, the variability in parental consent across centers (ranging from 16/30, 53% to 24/25, 96%) is worth noting. This variation may be owing to the differing relationships within centers between staff and parents regarding the contents of children’s lunchboxes with previous studies reporting a reluctance from staff to communicate with parents in fear of having difficult conversations [58,59]. As such, some parents may have been reluctant to consent to lunchbox measurements owing to perceived judgment [58,59]. Although not dissimilar to previous web-based studies conducted within the ECEC setting, the overall study consent rate among centers was moderate at 47% [27,60,61]. Similar to previous studies, barriers to center participation reported by staff included a lack of time and competing priorities [62]. As this study attempted to address such barriers by embedding the intervention within usual center processes (ie, aligning with ECEC accreditation standards), further consideration needs to be taken to better promote the intervention by aligning with current center priorities during study recruitment. However, once consented to the trial, the study data collection components were highly feasible, with 100% of participating centers completing child lunchbox measurements, center nutrition environment observations, and interviews with nominated supervisors. This indicates that such methods should be retained for a fully powered implementation trial.

Promising levels of uptake and acceptability of the implementation strategies used in this study were observed. The level of engagement with the web-based program was consistent with recommendations for centers to complete the self-assessment (audit with feedback) and develop action plans (formal implementation blueprint) twice during the intervention period. Such findings suggest that centers are likely to receive the intended dose of the intervention with the current implementation strategies. The promising levels of engagement may be attributed to the web-based program being easy to use as reported by nominated supervisors and aligned with usual center processes [63]. However, large SDs and wide IQRs for the number of log-ins and log-in duration indicate high variability in engagement with the web-based program across centers. Despite such variability being consistent with previous studies within the ECEC setting that used web-based modalities [27], exploration is needed to better understand the reasons behind the relatively lower levels of engagement for some centers.

As the intervention was largely delivered remotely, the overall cost to deliver the implementation strategies was minimal (total of Aus $ 1351.25 [US $ 931.88]; average per center: Aus $ 122.84 [US $ 86]). Therefore, the web-based intervention may be considered a low-cost alternative to support center implementation compared with traditional, highly intensive modalities. However, the study was unable to capture the costs associated with center staff implementing healthy eating practices, including the time spent disseminating information to parents. As such, future studies should consider conducting a cost-effective analysis, while capturing costs associated with center implementation of practices, to enable researchers, practitioners, funding bodies, and centers to determine whether investment in the web-based intervention produced an acceptable return and is a cost-effective approach to support the implementation of healthy eating practices at scale. Consistent with previous studies conducted within the ECEC setting [51,64], high levels of uptake and acceptability were found for most implementation strategies provided by HPOs, particularly the educational outreach visit (11/11, 100%) and local technical assistance (10/11, 91%). Despite previous literature suggesting that implementation strategies such as the MoU and center champions are useful for facilitating the uptake of interventions [19,39,44], the relatively low uptake of these strategies is worth exploring. Although there was high acceptability of the center champion strategy in centers that nominated a champion (9/10, 83%), a potential explanation for the lower uptake of the strategy may be the differing organizational structures within centers. Anecdotally, the uptake of center champions was higher in larger centers with greater staffing numbers and child enrollments, where the nominated supervisor often engages educational leads. The educational lead takes on additional advocacy roles among staff, lending them to the role of the center champion. In smaller centers however, the nominated supervisors often work as the educational lead themselves, acting as the main advocate among center staff. Therefore, the research team should consider alternative strategies, such as a local consensus approach [51] (ie, the entire center), to ensure that the uptake of the intervention remains high in centers where a sole champion is not a feasible strategy.

The improvement in implementation of 4 of the 5 targeted healthy eating practices within the intervention group is promising, with effect sizes ranging from 19% (2/11) to 64% (7/11). Such effect sizes are encouraging when compared with previous studies aimed at improving the implementation of practices within the ECEC setting [13]. A recent Cochrane systematic review, which examined the effectiveness of strategies aimed at improving the implementation of healthy eating and physical activity policies and practices, reported effect sizes as low as 2.5% [13]. Therefore, our findings show great promise for testing in a fully powered implementation trial. However, a decrease in centers supporting families to provide healthier foods consistent with dietary guidelines, particularly in those centers classified as most disadvantaged, is worth noting given this practice had the highest rates of implementation at baseline. A potential explanation for this reduction may be the competing information relating to COVID-19 distributed to parents during the intervention period (eg, communication regarding center safety protocols and changes to child attendance fees), resulting in support for parents to provide foods consistent with sector dietary guidelines being of lesser priority at this time. Research suggests a lack of skills, knowledge, and confidence in communicating with parents regarding healthy eating [58,59,65] may also negatively impact the implementation of this practice. Using strategies, such as ongoing professional development, coaching, and training, have been suggested in recent studies to address such barriers and support ECEC staff to engage in positive and effective communication with parents [65]. As centers were encouraged to distribute the healthy eating resources to parents via usual communication methods (eg, parent communication apps, email, and written information), further consideration of the most effective method to facilitate staff communication with parents regarding healthy eating and nutrition may be required. Although the Childcare EATS engagement data provided important insights into the center use of the web-based program, the methods used and the reach of the center distribution of healthy eating information and resources (eg, number of parents who received the resources) could not be measured. In addition, we were unable to assess whether parents within the intervention group communicated healthy eating information provided by the center staff to other parents. There was a notable contrast in the implementation of this practice between centers classified as most and least disadvantaged. This contrast may potentially be explained by COVID-19 related impacts on resourcing (eg, staffing, budget, and time) within disadvantaged centers, who may have already been experiencing limited resources before the pandemic. A better understanding of the barriers faced by centers classified as most disadvantaged in communicating with parents should be sought to enable the development of appropriate strategies to support implementation of this practice. However, given the small sample size in this study, this finding is highly exploratory and should be interpreted with caution. In addition, collecting contextual data from parents regarding their preferred method of receiving healthy eating information from centers may also provide guidance on the most effective way to support parents in packing healthy lunchboxes for children to consume in care.

The findings from this study provide compelling data to support the conduct of a fully powered implementation trial. Importantly, despite the relatively low level of support provided to childcare centers to use the program, the level of engagement with the web-based program was relatively high, and large changes in practice implementation were observed. Findings from this study suggest that several improvements could be made to the intervention, including considering the appropriateness of the MoU and center champion and using strategies to support ECEC center staff engagement with parents regarding healthy eating. Finally, the inclusion of a nested evaluation within a future trial to assess the impact of the web-based intervention on individual-level outcomes, including child dietary intake and parent lunchbox packing practices, should be considered to gain greater insight into the effectiveness of the intervention beyond center-level outcomes.

### Limitations

Although unavoidable because of restrictions relating to the COVID-19 pandemic, the inability to assess center nutrition environments and conduct child lunchbox assessments via direct observation to assess child-level outcomes as originally intended is a limitation of the study. In addition, although the data regarding the impact of the intervention on center implementation are promising, these data were only able to be collected within intervention centers with no comparison to the control group, and as such, should be interpreted with caution. Finally, as the study was conducted within 1 region of NSW, the generalizability of the findings beyond the region may be limited.

### Conclusions

This pilot study provides compelling data to support the conduct of a larger trial assessing the impact of the web-based intervention on ECEC center implementation of healthy eating practices. The findings of this pilot study indicate that the web-based intervention is highly feasible, acceptable, appropriate, and low-cost. As this study is one of few examining the potential impact of a web-based intervention within the ECEC setting, a fully powered implementation trial is warranted to establish the true effects and examine the impact of the intervention at scale.

## Appendix

Multimedia Appendix 1 CONSORT-eHEALTH checklist (V 1.6.1).

## Abbreviations

BCT: behavior change technique
BCW: Behavior Change Wheel
EATS: Electronic Assessment Tool and Support
ECEC: early childhood education and care
Go-NAPSACC: Go-Nutrition and Physical Activity Self-Assessment for Child Care
HNE: Hunter New England
HPO: health promotion officer
MoU: memorandum of understanding
NSW: New South Wales
RCT: randomized controlled trial
SES: socioeconomic status
